# Supplementary material for: Complexome profiling of the Chlamydomonas psb28 mutant reveals TEF5 as an early PSII assembly factor
Source: Plant Cell. 2025 Mar 17;37(6):koaf055. doi: 10.1093/plcell/koaf055 (PMC12164590; doi:10.1093/plcell/koaf055)
Supplement: koaf055_Supplementary_Data [file koaf055_supplementary_data.zip › psb28_interactive.zip!/plots/intensity/qualityPlots/peptides/IPB2_Cre14.g633750.t1.1.html]

### Cre14.g633750.t1.1

Trivial name: IPB2  
SeqCoverage [median]: 4.60  
Mass: 121.66 kDa  
  
 

| Sequence | MissedCleavage | Mass [Da] | wtIntensity | muIntensity | Score |
| --- | --- | --- | --- | --- | --- |
| ALLGAEPTAAAFK | 0 | 1258.69 | 0.00 | 770.72 | 54.61 |
| LQGAVPAETISGVVAK | 0 | 1538.87 | 0.00 | 881.56 | 61.38 |
| TYVAGPVGVR | 0 | 1017.56 | 0.00 | 441.04 | 51.85 |
| VAGAGDSLEPQTR | 0 | 1299.64 | 0.00 | 414.43 | 59.54 |
|  |  |  | 0.0 | 2507.8 |  |
